# Steatocystoma simplex in penile foreskin: a case report

**DOI:** 10.1186/s13256-016-0845-3

**Published:** 2016-03-08

**Authors:** Mário Maciel de Lima Jr, Mário Maciel de Lima, Fabiana Granja

**Affiliations:** 1 Department of Urology, Coronel Mota Hospital in Roraima State, Boa Vista - RR, Brazil; 2 Biodiversity Research Center, Federal University of Roraima (CBio/UFRR), Boa Vista - RR, Brazil; 3 Rua Levindo Inácio de Oliveira, 1547, Paraviana, Boa Vista, RR 69 307-272 Brazil

**Keywords:** Amazon, Foreskin, Indian, Penis, Steatocystoma

## Abstract

**Background:**

Steatocystoma simplex is an uncommon skin lesion with a histological pattern that is identical to that of steatocystoma multiplex. We are reporting this case of steatocystoma simplex for its uncommon location in the penile foreskin, and its occurrence in a Wapishana man.

**Case presentation:**

A 56-year-old man of Wapishana ethnicity presented with complaints of referred penile discomfort and pain during sexual intercourse for 5 years. A physical examination revealed a mobile, compressible subcutaneous non-tender mass of 4 cm diameter located on the left-side of his penile foreskin. There were no signs of inflammation, no grip on the penile shaft, and no urethral discharge or enlargement of lymph nodes. We found no evidence of other cysts on cutaneous examination. We performed classical excision of the lesion under local anesthesia and confirmed the diagnosis of steatocystoma with the pathological report. As there were no complications, we discharged him the same day.

**Conclusion:**

Steatocystoma can be considered a differential diagnosis for cystic lesions on and around the penis.

## Background

Steatocystoma simplex is a rare skin lesion with a histological pattern that is identical to that of steatocystoma multiplex. Steatocystoma multiplex is a condition with multiple cystic lesions inherited in an autosomal dominant pattern. However, steatocystoma is a non-inheritable solitary lesion. Brownstein, in 1982, was the first to describe steatocystoma simplex [1]. Since then, there have been only 10 reports in the English literature up to 2013. Steatocystoma simplex cysts usually occur on the face, trunk and extremities [2]. To the best of our knowledge, there are no reports on steatocystoma simplex on the penile foreskin or on people of Wapishana ethnicity. Hence, we are presenting this case report on steatocystoma simplex for its rare location on penile foreskin, and its occurrence in a Wapishana man.

## Case presentation

**Fig. 1:**
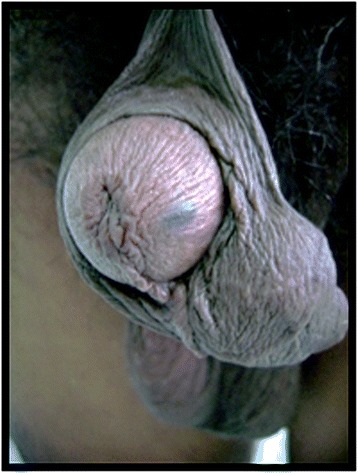
View of the mass located on the left-side penile foreskin from front

**Fig. 2:**
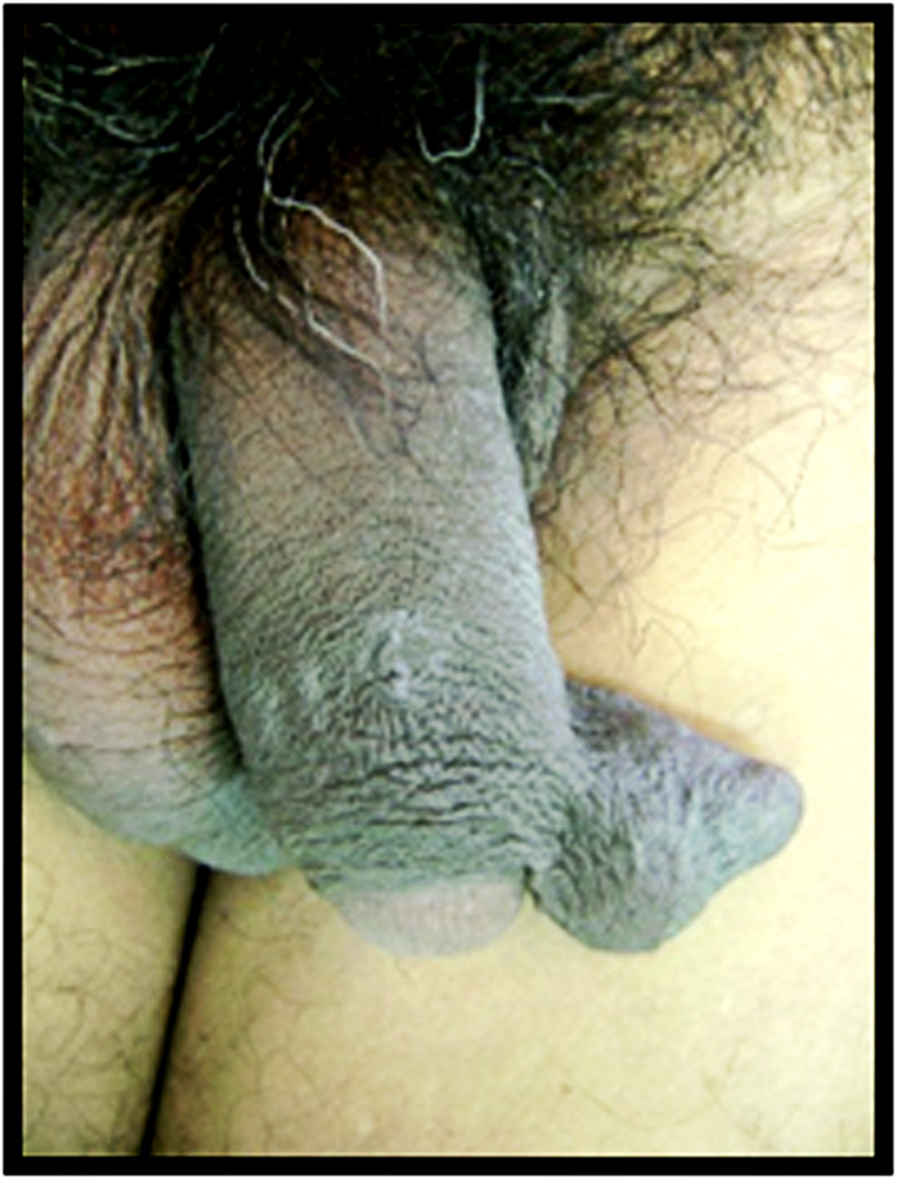
View of the mass located on the left-side penile foreskin from above

**Fig. 3:**
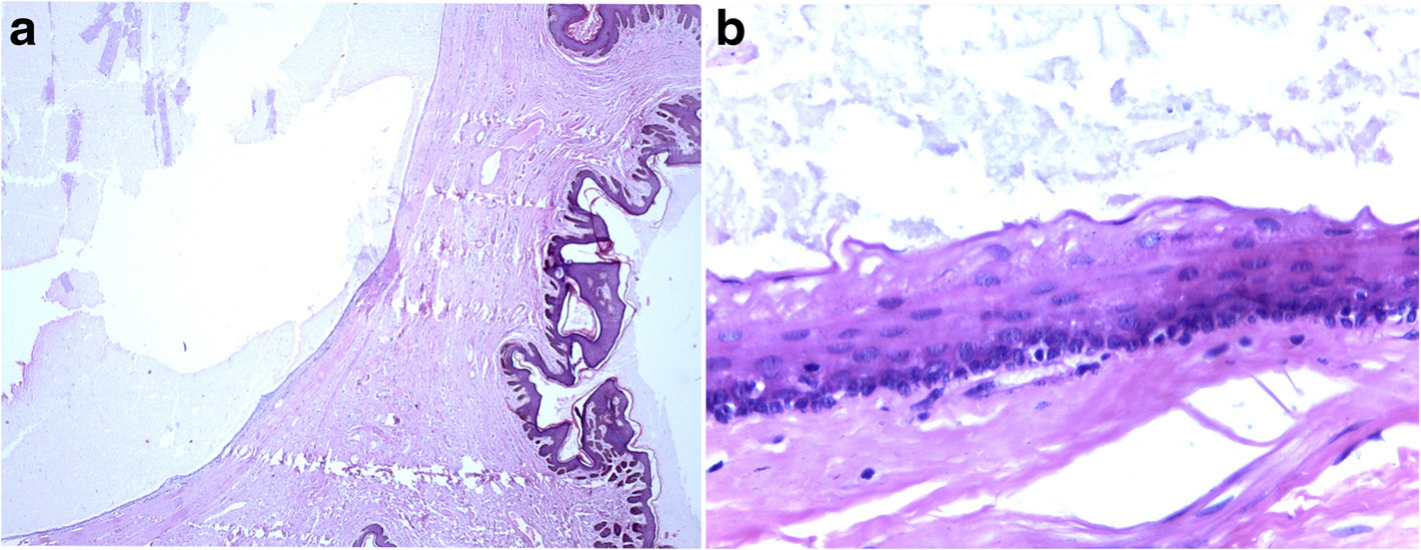
Classical excision of the lesion with local anesthesia and diagnosis of steatocystoma with the pathological report (**a**, **b**)

A 56-year-old Indian man of Wapishana ethnicity presented to our Surgery out-patient department (OPD) with complaints of referred penile discomfort and pain during sexual intercourse for 5 years. He was referred to our Urology department as a case of genital tumor. A physical examination revealed a mobile, non-tender, compressible, subcutaneous mass of 4 cm diameter located on the left-side of his penile foreskin (Figs. 1 and 2). He had noticed the lesion 5 years before and the lesion gradually increased in size. There were no signs of inflammation over the skin covering the cyst. There was no grip on the penile shaft; there was no urethral discharge or enlargement of spermatic cord or inguinal lymph nodes. We found no evidence of any other cysts on cutaneous examination of his entire body. His nails, hair and teeth were normal. There was no family history of a similar condition. He had no history of hypertension, diabetes or any chronic illness and was not under any medication. We performed a classical excision of the lesion with local anesthesia and confirmed the diagnosis of steatocystoma with the pathological report (Fig. 3a, b).

On histopathological examination, the cyst consisted of numerous folds lined by stratified squamous epithelium. A basal layer, two or three rows of pavement cells, and a well-defined corneal layer that protruded inside the cyst, simulating the beheading of apocrine glands, formed the cyst wall. Sebaceous glands were also found within the cyst wall itself.

There was no infection or pain after excision. The patient was discharged from hospital the same day, after surgery.

## Discussion

Steatocystoma is a benign adnexal tumor that originates from the pilosebaceous duct. It usually presents with multiple lesions, but Brownstein [1] reported that this entity could manifest as isolated lesions. We encountered a rare case of steatocystoma simplex in the penile foreskin, and we are the first to report a case of steatocystoma simplex in Wapishana people; an indigenous group of people found in southern Guyana and northern Brazil. It usually manifests as a cystic lesion of 1 to 1.5 cm diameter in the face, trunk and extremities [2] and lesions greater than 2 cm are uncommon. The present lesion was 4 cm in diameter, which is also a rare presentation.

Steatocystoma simplex is a benign, solitary, non-heritable counterpart of steatocystoma multiplex that is inherited in an autosomal dominant fashion with multiple lesions [1]; steatocystoma simplex and steatocystoma multiplex have identical histological patterns. We ruled out steatocystoma multiplex after careful examination of our patient’s entire body and found no evidence of papules or nodular lesions other than his penile cystic lesion. Steatocystoma simplex is generally reported in areas of the neck, nose, armpit, back, legs, scalp and chest. Even though steatocystoma multiplex cysts are commonly seen in the penile and scrotal region steatocystoma simplex’s presentation in penile foreskin has not been reported until now.

Cystic lesions in and around the penis are uncommon. Cystic lesions that have been reported until now are: mucoid cysts, epidermal cysts, pilonidal cysts, Tyson gland cysts and urethral diverticulum [3–9]. Dermoid cyst was our first differential diagnosis since it also contains pilosebaceous structures opening into the cyst. The main difference between dermoid cyst and steatocystoma is the presence of hair shafts inside the dermoid cyst. Median raphe cysts/mucoid cysts are congenital lesions that arise due to sequestered ectopic urethral mucosa during embryological development. They can develop only along the median raphe of the male external genitalia [10]. The cyst in this case was not on the midline but on the left-side foreskin. Tyson gland cysts are painful and commonly occur after an infection. The cyst in our patient was non-tender, without any signs of inflammation or urethral discharge. Epidermal inclusion cyst in penis usually arises because of proliferation of implanted epidermal elements within the circumscribed space of the dermis. There was no circumcision or any surgery of the penis to implant epidermal elements in our patient. One of the major limitations was that no computed tomography (CT) or magnetic resonance imaging (MRI) was performed to analyze various differential diagnoses. The confirmation of diagnosis was only through the pathological report and clinical examination.

Steatocystoma simplex cysts are non-symptomatic, and the treatment is done usually for cosmetic purposes. The indications for the treatment in the present case were pain and discomfort during sexual intercourse, and cosmetic disfigurement. We performed classical excision of the lesion under local anesthesia. As there were no complications, we discharged our patient the same day. Caution is necessary when treating steatocystoma simplex because aspiration, disruption, or partial excision of the cyst may lead to recurrence. The cyst should be removed intact. Uneventful postoperative recovery without any complication suggests surgery is the best option to manage such tumors. Hence, we recommend resection of such tumors as the best possible management.

## Conclusion

Steatocystoma can be considered a differential diagnosis for cystic lesions on and around the penis.

## Consent

Written informed consent was obtained from the patient for publication of this case report and accompanying images. A copy of the written consent is available for review by the Editor-in-Chief of this journal.
